# Supplementary material for: Identification of photoperiod-induced specific miRNAs in the adrenal glands of Sunite sheep (Ovis aries)
Source: Front Vet Sci. 2022 Jul 22;9:888207. doi: 10.3389/fvets.2022.888207 (PMC9354845; doi:10.3389/fvets.2022.888207)
Supplement: Supplementary file 1 [file Data_Sheet_1.ZIP › Supplementary file/miRDeep2 file.zip!/miRDeep2 file/Know_Structure/SP42A1/oar-mir-381.pdf]

miRBase precursor : oar-mir-381
Total read count : 1232
oar-miR-381-5p read count : 2
oar-miR-381-3p read count : 1230
remaining reads : 0

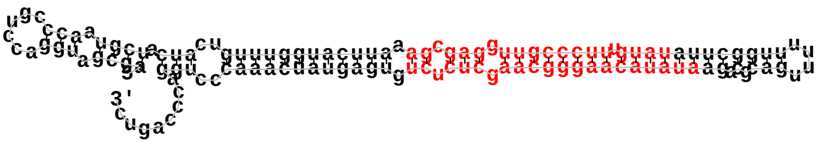

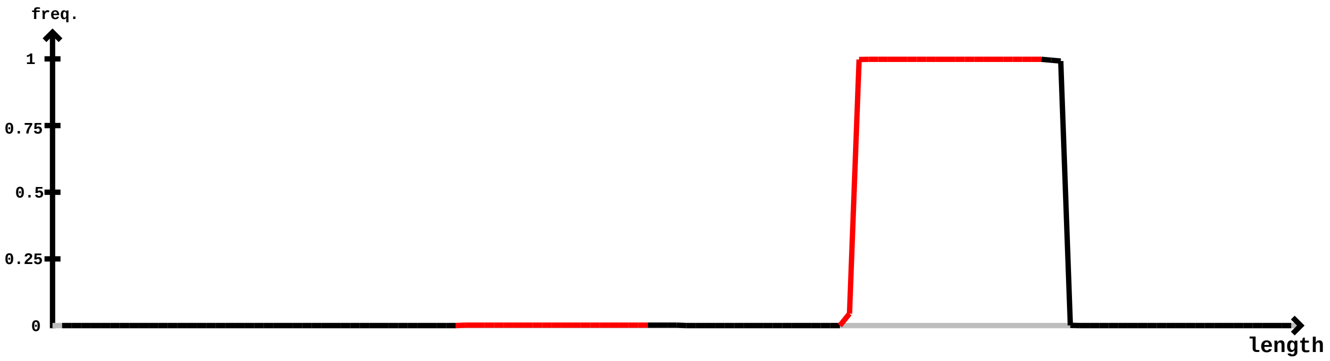

oar-miR-381-3p

oar-miR-381-5p

```
5'- agcgauggaccugcccaaugcuacuacuguuugguacuuaaagcgagguugcccuuuguauauucgguuuuuugacgcagaauauacaagggcaagcucucugugaguaucaaacccuggacccaguc -3'    exp
    ((((.(((......))).)))).(((..((((((((((((.((.(((.((((((((.((((((((((((....))).).))))))))))))))))).))).)).))))))))))))..)))........       reads     mm
    .........................................agcgagguugcccuuuguauauu................................................................       2         0
    .................................................................................auauacaagggcaagcucucu..........................       1         0
    .................................................................................auauacaagggcaagcucucugu........................       54        0
    ..................................................................................uauacaagggcaagcucucu..........................       3         0
    ..................................................................................uauacaagggcaagcucucug.........................       4         0
    ..................................................................................uauacaagggcaagcucucugu........................       1166      0
    ..................................................................................uauacaagggcaagcucucugug.......................       1         0
    ...................................................................................auacaagggcaagcucucugu........................       1         0
```
